# Effect of Heat Treatment on the Microstructure and Performance of Cu Nanofoams Processed by Dealloying

**DOI:** 10.3390/ma14102691

**Published:** 2021-05-20

**Authors:** Jenő Gubicza, Péter Jenei, Gigap Han, Pham-Tran Hung, Youngseok Song, Dahye Park, Ábel Szabó, Csilla Kádár, Jae-Hun Kim, Heeman Choe

**Affiliations:** 1 Department of Materials Physics, Eötvös Loránd University, P.O. Box 32, H-1518 Budapest, Hungary; jenei@metal.elte.hu (P.J.); hungpthung95@gmail.com (P.-T.H.); abel.szabo@ttk.elte.hu (Á.S.); kadar@eik.bme.hu (C.K.); 2 School of Materials Science and Engineering, Kookmin University, 77 Jeongneung-ro, Seongbuk-gu, Seoul 02707, Korea; hgg4602@gmail.com (G.H.); sollabim2@gmail.com (Y.S.); dahyepark@kookmin.ac.kr (D.P.); jaehunkim@kookmin.ac.kr (J.-H.K.); heeman@kookmin.ac.kr (H.C.); 3 Department of Materials Science and Engineering, Budapest University of Technology and Economics, Műegyetem Rakpart 3, H-1111 Budapest, Hungary; 4 MTA-BME Lendület Composite Metal Foams Research Group, Műegyetem Rakpart 3, H-1111 Budapest, Hungary

**Keywords:** dealloying, Cu nanofoam, heat treatment, microstructure, hardness

## Abstract

Cu nanofoams are promising materials for a variety of applications, including anodes in high-performance lithium-ion batteries. The high specific surface area of these materials supports a high capacity and porous structure that helps accommodate volume expansion which occurs as batteries are charged. One of the most efficient methods to produce Cu nanofoams is the dealloying of Cu alloy precursors. This process often yields nanofoams that have low strength, thus requiring additional heat treatment to improve the mechanical properties of Cu foams. This paper provides the effects of heat treatment on the microstructures, mechanical properties, and electrochemical performance of Cu nanofoams. Annealing was conducted under both inert and oxidizing atmospheres. These studies ultimately reveal the underlying mechanisms of ligament coarsening during heat treatment.

## 1. Introduction

Nanofoams are foams with pore sizes that range from several to hundreds of nanometers [1]. These materials have several important applications, including catalysts, fuel cells, substrates for heat-exchanger applications, sensors, actuators, dye-sensitized solar cells, microfluidic flow controllers and anodes in high-performance lithium-ion batteries [2,3,4,5,6]. In the latter case, the very high specific surface area of nanofoams can promote high battery capacities. Moreover, the pores in nanofoam materials can help to accommodate the volume changes that occur during the charging cycle of batteries, as one of the fundamental reasons for the electrode material’s volume change, fracture/cracking and capacity degradation could be the diffusion-induced stresses [7,8,9,10]. Nonetheless, in some applications, such as in gas diffusion layers [11] and electrodes in polymer electrolyte membrane fuel cells [12], the coexistence of nano- and micron-sized pores can further improve the performance of the foam because the micropores facilitate the flow of gas and water.

Metallic nanofoams can be processed by chemical dealloying of single-phase solid–solution binary alloys [13]. This technique leaches minimal amounts of noble metals out of the alloy, so more noble metal remains in the nanoporous material (selective dissolution). For example, aluminum can be dealloyed from an Al–Cu system under either acidic (e.g., HCl) or alkaline (e.g., NaOH) solutions, yielding a Cu nanofoam because the standard potential of Al is much lower than that of Cu [14]. Bulk Al–Cu intermetallic compounds produced by techniques such as powder metallurgy methods [15,16] or pack cementation technique [17] serve as precursors for materials with nanosized pores. As an example, an Al_2_Cu phase generated by pack cementation can be easily dissolved by an aqueous solution of HCl [17]. By comparison, other phases (e.g., Al_4_Cu_9_) cannot be dissolved. This dissolution behavior necessitates the use of materials with chemical compositions of around 67 at.% Al and 33 at.% Cu to process Cu nanofoams. Moreover, the pack cementation time also has a significant bearing over the phase composition of the bulk material and, therefore, on the pore structure of the resulting dealloyed Cu foam. During pack cementation, a Cu plate is placed in a bed of Al powder, heated above the melting point of aluminum, and maintained at this temperature for different periods [18]. During this heat-treatment process, the Al particles melt, and Al atoms diffuse into the Cu to form an Al_2_Cu phase. At long pack cementation times (>12 h), an Al phase also forms in addition to and alongside the Al_2_Cu intermetallic compound [18]. Subsequent dissolution of such aluminum products yields materials with micron-sized pores in place of the Al phases, resulting in a hierarchical pore structure comprising both micro- and nano-pores.

Furthermore, the internal surfaces of dealloyed Cu foams can be replete with an “active” layer that stores Li-ions, which is beneficial for battery applications. These coatings can be composed of different materials such as tin or copper-oxide [17,19,20]. Annealing processes under oxidizing environments at different temperatures can tailor the structures (e.g., CuO versus Cu_2_O), morphologies, and thicknesses of the surface oxide layer, thereby influencing the electrochemical performances of the nanofoam materials [21]. Heat treatments conducted under inert atmospheres do not modify surface oxides but can substantially improve the mechanical strengths of the dealloyed Cu foams [22]. This enhanced mechanical reliability of the Cu nanofoam can then allow it to be used in the form of a thin, large-area sheet, thus providing a considerably wider range of practical applications. For example, the strengthened Cu nanofoam can then be used as an advanced functional material for battery electrodes, catalysts, heat exchangers, and filters by enabling efficient and rapid electrochemical reactions owing to their high specific surface area [23].

In this study, the effects of thermal annealing under inert and oxidizing atmospheres are investigated. The ligament coarsening, and the change of the mechanical and electrochemical performances due to the heat treatments, are discussed. Moreover, this study presents a summary of literature data in this field, including recent research results.

## 2. Materials and Methods

### 2.1. Processing of Cu Foams and the Annealing Conditions

The influence of heat treatments on ligament sizes in Cu nanofoams prepared by dealloying was investigated in samples processed by three distinct routes (routes A–C). For all specimens, the bulk precursor materials were obtained by pack cementation. In route A, a blend of Al, Al_2_O_3_, and NH_4_Cl powders were mixed and stacked on a Cu disk with a diameter of 11 mm (which is a common size for a 2032-type coin cell) and thickness of 0.25 mm in a stainless-steel container. This powder blend with the Cu plate was heat-treated at 800 °C for 6 h (the pack-cementation step) in a box furnace (Korea Furnace Development Co., Yangju, South Korea). During the pack cementation process, Al atoms diffused into the Cu plate as a result of the NH_4_Cl powder serving as a chemical reaction activator at elevated temperatures. The result of this process is an Al_2_Cu phase [17]. The Al_2_O_3_ filler in the powder blend slows the process and enables control over the uniformity of Al layer on Cu by decreasing the effective surface of the Cu disk [17]. After the pack-cementation process, the material was annealed at 700 °C for 9 h and subsequently at 500 °C for an additional 6 h under an Ar atmosphere, which ensured a homogeneous distribution of Al in the Al_2_Cu phase. The pack-cemented bulk sample was mechanically ground by using abrasive grinding SiC papers (with 1000, 2500, and 4000 grit sizes) and polished by using a 1 µm Al_2_O_3_ suspension. After surface treatment, the pack-cemented bulk sample was cut into pieces, dealloyed and used for additional annealing. For samples prepared by route A, dealloying was conducted by etching in an aqueous solution of 2 wt.% HCl at 45 °C for 12 h. The dealloyed sample was further annealed at 400 °C for 6 h. For samples prepared by route B, the conditions of pack cementation were similar to those of route A except that the pack cementation time was increased to 15 h. The longer pack-cementation time promotes higher Al content, so an additional Al phase forms in tandem with Al_2_Cu. The presence of the Al phase is significant because its dissolution during the dealloying process provides large pores in the resulting Cu foam (5–6 µm) in addition to the nanopores, which have sizes of less than 100 nm [18]. Thus, such materials have hierarchical pore structures. In route B, dealloying was conducted in an aqueous solution of 5 wt.% HCl at 90 °C for 12 h. The Cu foams processed by route B were heat-treated under two different conditions, namely 300 °C for 70 h and 800 °C for 5 h. In route C, all pack cementation and dealloying conditions were identical to route B; however, the concentration of HCl was 10 wt.% instead of 5 wt.%. The Cu foams processed by route C were heat-treated under three different conditions: 300 °C for 70 h, 600 °C for 5.5 h, and 800 °C for 5 h.

The effect of annealing under oxidizing atmospheres on the microstructure and electrochemical behaviors of Cu foams processed by dealloying was also investigated. In these experiments, the initial sample was processed by route A. Subsequent annealing in air was conducted at 110, 140, 170, and 200 °C for 30 min to grow oxidation layers that were used as an anode active material in Li-ion batteries.

### 2.2. Study of the Microstructure

The ligament sizes of the dealloyed and annealed Cu foams were determined by scanning electron microscopy (SEM) using an FEI Quanta 3D electron microscope (manufacturer: Thermo Fisher Scientific, Waltham, MA, USA). The acceleration voltage and the vacuum were 20 kV and 10^−4^ Pa, respectively. The average ligament size was obtained using a standard metallographic method in which straight lines were placed randomly on the images, and the average length of the segments of the straight lines lying inside the ligaments was determined. The measurement for ligament size was carried out for as many ligaments as possible. Depending on the ligament size and the amount of SEM figures taken, the number of ligaments used to determine the ligament size was between 20 and 180.

The microstructure inside the Cu ligaments was studied by X-ray line profile analysis (XLPA) which is a non-destructive method to determine the density of dislocations and twin faults [24]. Moreover, XLPA interrogates much larger volumes than microscopic methods, such as transmission electron microscopy (TEM), and thereby provides a complementary and quantitative bulk characterization of the lattice defect structures. The X-ray line profiles were measured with a RA MultiMax-9 high-resolution rotating anode diffractometer (manufacturer: Rigaku, Tokyo, Japan) using CuKα_1_ radiation (wavelength: 0.15406 nm). One pattern was taken for each foam. A quantitative evaluation of diffraction peaks was performed by applying the convolutional multiple whole profile (CMWP) fitting method [25]. In this procedure, the measured X-ray data are approximated by the sum of a background spline and subsequently fit to theoretical diffraction profiles of model microstructures, which contains the various defining parameters of the microstructure such as the average crystallite size, dislocation density, and twin fault probability. The twin fault probability in face-centered cubic (fcc) crystals is defined as the fraction of twin faults among the {111} lattice planes. It should be noted that the phrase “crystallite” refers to the underlying crystalline structure inside the ligament, while the word “ligament” refers to the morphological features of the foam.

### 2.3. Characterization of the Mechanical Behavior by Nanoindentation

The influence of annealing on the mechanical behaviors of Cu foams processed by route C was investigated using nanoindentation. Approximately 20 Vickers tests were conducted on each foam sample before and after annealing at 600 °C for 5.5 h. A UMIS nanoindentation device with Vickers indenter tip was used (manufacturer: CSIRO, West Lindfield, Australia), applying a maximum load of 2 mN. The hardness and the elastic modulus values were determined by applying the Oliver–Pharr method [26].

### 2.4. Study of the Electrochemical Performance of the Cu Foams Annealed under Oxidizing Atmospheres

To demonstrate the performance of the synthesized Cu nanofoam as a LIB anode current collector, a Cu oxide layer, a high-capacity anode active material, was thermally grown on the surface of Cu nanofoam in an air tube furnace (DBP-6P, Dong-Jin Machine, South Korea). All of the Cu/Cu oxide nanofoam electrodes were prepared with a dimension of 11 mm in diameter and 250 μm in thickness. CR2032-type coin-cells were assembled in a glove box in a dry Ar atmosphere using the Cu/Cu oxide nanofoam anode coupon as the working electrode and a Li metal foil for both the counter and reference electrodes. The electrolyte was a traditional 1 M LiPF_6_ solution of ethylene carbonate (EC) and diethylene carbonate (DEC) in a 3:7 volume ratio. Galvanostatic tests (WBCS3000 cycler, WonATech, South Korea) were carried out on the assembled coin cells containing the Cu/Cu oxide nanofoam anode at a current density of 1 mA/cm^2^ in the voltage range of 3.0 V to 0.01 V (vs. Li-ion/Li) at 25 °C.

## 3. Results and Discussion

### 3.1. Effects of Annealing under Inert Gas Atmospheres on the Microstructural and Mechanical Properties

#### 3.1.1. Ligament Coarsening Due to Heat Treatment

**Figure 1:**
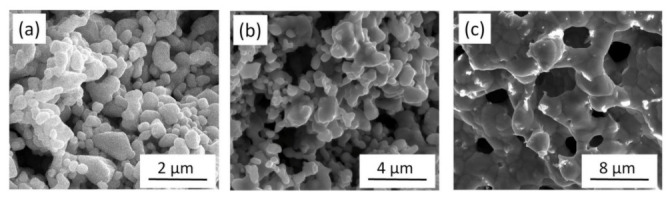
Scanning electron microscopy (SEM) images for Cu foams (**a**) processed by using route B and annealed at (**b**) 300 °C for 70 h and (**c**) 800 °C for 5 h.

**Figure 2:**
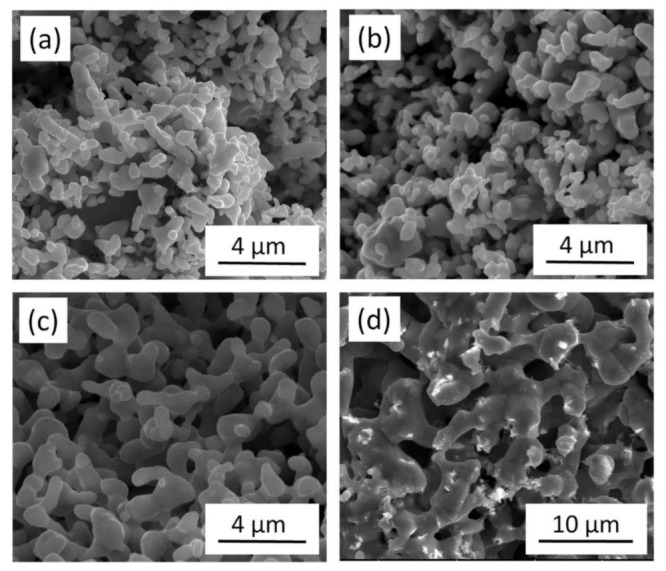
SEM images of (**a**) Cu foam materials processed by route C and subsequently annealed at (**b**) 300 °C for 70 h, (**c**) 600 °C for 5.5 h (**d**) and 800 °C for 5 h.

Figure 1a shows the SEM image of the Cu nanofoam prepared using route B, demonstrating an average ligament size of approximately 640 nm. Annealing at 300 °C for 70 h and 800 °C for 5 h resulted in ligament coarsening, as revealed in the SEM images in Figs. 1b and c. The average ligament sizes for materials annealed at 300 °C for 70 h and at 800 °C for 5 h were ~770 and ~5180 nm, respectively (see also Table 1). Similar ligament size was obtained for route C; the initial ligament size for the dealloyed foams varied between 540 and 740 nm. This demonstrated that changing the HCl concentration from 5 to 10 wt.% did not influence the ligament size significantly (see Figure 2a and Table 1). It is noted that for route C the ligament sizes after dealloying differ slightly in different samples (540 and 740 nm). This observation can be explained by the fact that the samples with the ligament sizes of 540 and 740 nm were prepared from two different pack cemented disks. X-ray diffraction revealed that the phase composition (the fractions of Al and Al_2_Cu phases) and the crystallographic texture of the main Al_2_Cu phase were different in the two disks. This effect can cause the difference between the ligaments’ sizes after dealloying. Annealing at 300 °C for 70 h, 600 °C for 5.5 h, and 800 °C for 5 h produced materials with ligament sizes of ~980, ~1620, and ~4220 nm, respectively, as shown in Figure 2b–d and Table 1. The shorter pack cementation time and the lower temperature of dealloying used for route A resulted in much smaller ligament sizes of ~105 nm, which increased to ~125 nm in materials prepared using heat treatment at 400 °C for 6 h (the corresponding SEM images are shown in [22]).

The kinetics of microstructure coarsening during annealing of foams has been described by the following relationship [27,28,29]:

(1) $$d^{n}-d_{0}^{n}=t\cdot K\left(T\right)$$

where *d* is the ligament size after heat treatment, *d_0_* is the size before heat treatment, *T* is temperature, *t* is time, and exponent *n* may vary between 3 and 4 [29], depending on the mechanism of coarsening. *K*(*T*) can be expressed by the following Equation:

(2) $$K\left(T\right)=\frac{k}{T}e^{-Q/RT}$$

where *k* is a constant, *Q* is the activation energy of the mechanism of coarsening, and *R* is the universal gas constant. The formulas presented above are also used to describe Ostwald ripening in bulk materials [30]. It is well understood [31,32,33,34,35] that the exponent *n* allows discrimination between a coarsening operated by surface diffusion (*n* = 4) and lattice diffusion (*n* = 3). A previous study on Au foams showed [35] that *n* was closer to 4, indicating that the surface diffusion tends to dominate over lattice diffusion. On the other hand, the experimentally determined activation energy (*Q*) for an Au foam suggests that lattice diffusion might also be a dominant coarsening mechanism if the annealing is performed in a vacuum [33]. The activation energy of the underlying mechanism of ligament growth can be obtained from the slope of the straight line fitted to the plot of $ln\left\{\left[d^{n}-d_{0}^{n}\right]T/t\right\}$ versus 1/*T*. A former study [29] also shows that the accuracy of fitting is similar either for *n* = 3 or 4 in the case of Au nanofoams. Herein, we thus carried out the analysis for both exponents.

**Figure 3:**
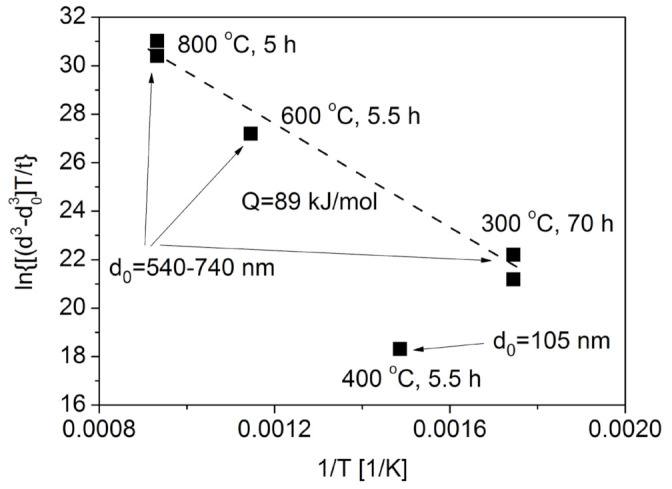
A plot of $ln\left\{\left[d^{3}-d_{0}^{3}\right]T/t\right\}$ versus 1/T used to determine the activation energy of the mechanism behind ligament coarsening in Cu foams processed by dealloying and subsequently annealed under an inert atmosphere. The plotting was performed based on Equation (3). The grain sizes (*d* and *d*_0_) were taken in nanometres, while the units of t and T were hours and Kelvin degrees, respectively.

As an example, Figure 3 shows a plot of $ln\left\{\left[d^{n}-d_{0}^{n}\right]T/t\right\}$ versus 1/*T* for the six Cu foams listed in Table 1 using *n* = 3. The data can be fitted well by a straight line for the samples processed by routes B and C, while the datum point related to the specimen produced by route A does not follow this trend. The same observation was found for *n* = 4 (not shown here). This deviation can be explained by the much smaller ligament size for the foam processed by route A. Indeed, routes B and C resulted in initial ligament sizes between 540 and 740 nm, while the foam processed by route A had a much smaller ligament size (about 105 nm). Unlike in bulk samples, in the case of foams, the material for coarsening is supplied by diffusion along the ligaments. Therefore, the diffusion rate is influenced by the thickness of ligaments since their interior and/or surface serve as paths for material flow. Namely, the larger the ligament size, the faster the diffusion. Thus, for smaller ligament size the coarsening is less pronounced under the same heat treatment conditions (time and temperature). This is the reason why the foam having an initial ligament size of about 105 nm does not follow the trend suggested by the samples with the ligament sizes between 540 and 740 nm (see Figure 3).

**Figure 4:**
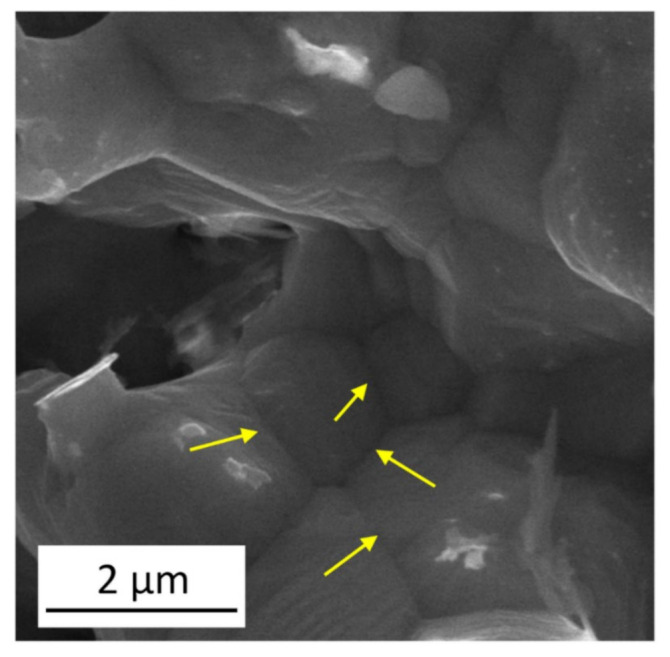
SEM image illustrates several ligaments grown in the material processed by route C and subsequently annealed at 800 °C for 5 h. The yellow arrows indicate boundaries between the coalesced ligaments.

The accuracy of fitting for the plot of $ln\left\{\left[d^{n}-d_{0}^{n}\right]T/t\right\}$ versus 1/*T* was similar for *n* = 3 and 4 as shown from the correlation coefficients listed in Table 2. The fitting on the datum points related to dealloyed Cu foams having initial ligament sizes of 540–740 nm yields an activation energy of about 89 ± 5 and 103 ± 11 kJ/mol for *n* = 3 and 4, respectively. Thus, both evaluations gave practically the same activation energy. This value is approximately half of the activation energy of the self-diffusion of Cu (210 kJ/mol [36]), being close to the activation energy of Cu diffusion along grain boundaries and dislocations. Indeed, the activation energy of diffusion varies with the nature of the grain boundaries, assuming the values between 81 and 104 kJ/mol [37,38,39]. It should be noted that the rate of diffusion along dislocations is similar to that in the grain boundaries and in the next section it will be revealed that the dealloyed Cu foams contain dislocations with a large density. However, it is worth noting that the activation energy values determined experimentally in this study fall within the range determined for surface diffusion in Cu. Indeed, former investigations revealed that the migration of Cu atoms on the surface of Cu can occur by either bridge diffusion of adatoms or a vacancy exchange mechanism. For the adatom and vacancy migration processes, the activation energy values are in the ranges of 3–124 and 27–114 kJ/mol, respectively [40,41,42]. Therefore, both diffusions along the defects (e.g., boundaries and dislocations) inside the ligaments and on the surface can contribute to ligament coarsening in the Cu foams investigated in this study. Our conclusion is that the growth of ligaments is controlled by a diffusion that is faster than bulk diffusion. The SEM image in Figure 4 shows several ligaments grown in the material processed by route C and annealed at 800 °C for 5 h. Arrows indicate boundaries between the ligaments, which may serve as paths for fast diffusion occurring during coarsening.

#### 3.1.2. Changes in Defect Density during Annealing

**Figure 5:**
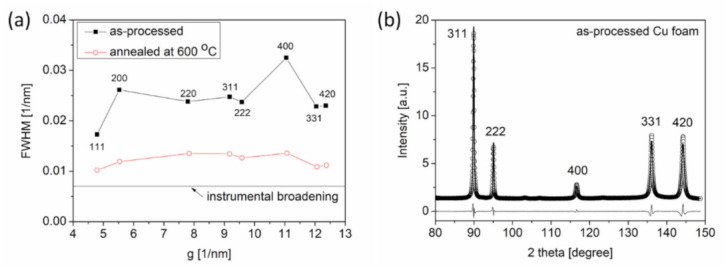
(**a**) Williamson–Hall plots for Cu foam processed by route C and the counterpart of this material, which was annealed at 600 °C for 5.5 h after dealloying. FWHM: full width at half maximum, g: magnitude of the diffraction vector (see text). (**b**) A section of the diffraction pattern evaluated by the CMWP fitting method using data obtained from a Cu foam processed by route C. The open circles represent the measured data, and the solid line reflects the fitted pattern. The difference between the data and the calculated pattern is shown as a black line at the bottom of the figure.

Former studies show that the ligaments in dealloyed Cu foams contain a considerable density of lattice defects, such as dislocations and twin faults [18,22]. These defects are grown during foam processing. Post-processing annealing significantly influences the defect density. Qualitative effects of annealing on the microstructure can be obtained by inspecting the XRD peak breadth. Figure 5a shows the classical Williamson–Hall plots for the Cu foam processed by route C and its counterpart that was annealed at 600 °C for 5.5 h after dealloying. This graph shows the full width at half maximum (*FWHM*) values of the diffraction peaks plotted against a function of the magnitude of the diffraction vector, which is denoted as *g* [24]. The *FWHM* and *g* are obtained using cos*θ*·Δ(2*θ*)/*λ* and 2·sin*θ*/*λ*, respectively, where *θ* is the Bragg angle, Δ(2θ) is the peak breadth, and λ is the wavelength of the X-ray. Analyses of Figure 5a reveal that the heat treatment at 600 °C for 5.5 h produced a significant reduction in the peak breadth for the Cu foams processed by route C. Notably, the widths of the diffraction peaks were larger than instrumental broadening even after annealing at 600 °C for 5.5 h, which indicates the presence of a significant defect density in the microstructure.

Figure 5b illustrates the CMWP fitting on the diffraction pattern acquired from Cu foam processed by route C. Only a section of the diffractogram is shown in this figure to provide better visibility of the coincidence between the measured and calculated patterns. The crystallite size, dislocation density, and twin fault probability obtained by XLPA for two dealloyed Cu foams processed by routes A and C are listed in Table 3. While the processing route affects the microstructural parameters of the resulting foam, the values of these parameters are on the same order of magnitude. For example, the crystallite sizes are a few tens of nanometers for both materials, and the twin fault probability is 1–2% different across the two materials, while the dislocation densities for both materials are 10^14^ − 10^15^ m^−2^. Differences between the defect structures can also be observed for Cu foams processed under the same nominal pack cementation and dealloying conditions. The dislocations and twin faults in the Cu foams are as-synthesized defects that form during the development of Cu ligaments over the course of the dealloying process.

**Figure 6:**
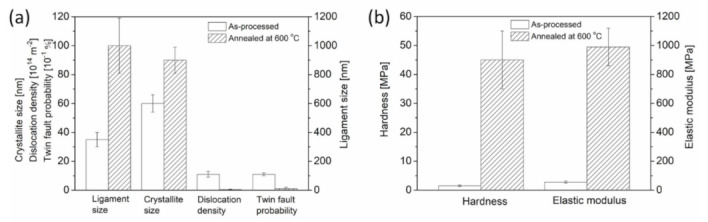
Comparison of (**a**) microstructural parameters and (**b**) hardness and elastic modulus obtained via indentation measurements for Cu foams processed by route C, as well as its counterpart material that was annealed at 600 °C for 5.5 h after dealloying.

While there are variations in the microstructural parameters of foams prepared by different processing routes, heat treatment causes significant changes in the defect structure of both dealloyed Cu foams, as shown in Table 3. Namely, the crystallite size increased while the twin fault probability decreased during annealing. Moreover, the dislocation density of the foam processed by route C decreased by more than one order of magnitude during heat treatment at 600 °C for 5.5 h. By comparison, considerable changes in the dislocation density did not occur in the Cu foam processed by route A when the sample was annealed at 400 °C for 5.5 h. Dislocations most likely form at the interfaces between ligaments where the mismatch stresses between the Cu crystallites are reduced by developing dislocations. The different structures and morphologies of the ligaments in the foams processed by the two routes cause the dislocation densities in the as-processed samples to be different. For materials prepared by route A, the dislocation density is lower and did not change during heat treatment, most likely due to the relatively low temperature of annealing. For samples processed by route C, the comparatively higher annealing temperature resulted in a pronounced reduction of lattice defects, as shown in Table 3. Changes in the microstructural parameters for this sample are also presented in Figure 6a. Importantly, the ligament size determined by SEM differs from the crystallite size obtained by XLPA. This discrepancy arises because the latter method is very sensitive to small misorientations. Thus, the fact that the crystallite size is considerably smaller than the ligament size suggests that the ligaments fragment into smaller regions with various crystallographic misorientations. The decrease of the defect density during annealing of the dealloyed Cu foams certainly softens the ligaments. However, the coalescence of the ligaments during heat treatment produces a strengthening effect. Thus, understanding the overall result of these two opposing effects during annealing is an important objective for future studies.

#### 3.1.3. Effect of Heat Treatment on the Mechanical Properties of Cu Foams

**Figure 7:**
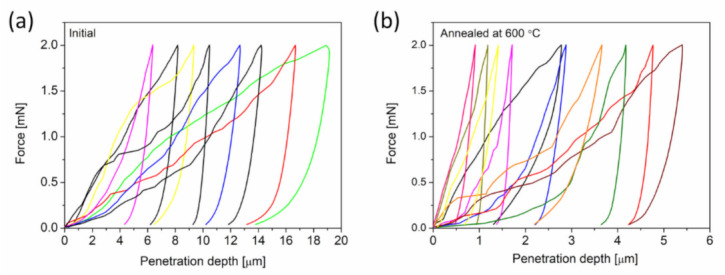
Force versus penetration depth curves obtained by nanoindentation from Cu foam materials that were processed by route C (**a**) and the counterpart material that was annealed at 600 °C for 5.5 h after dealloying (**b**).

The influence of annealing on the mechanical behaviors of Cu foams processed by route C was investigated by using nanoindentation. Approximately 20 Vickers tests were conducted on each foam before and after annealing at 600 °C for 5.5 h. Representative force-penetration depth curves for the as-processed and heat-treated materials are shown in Figure 7a,b, respectively. High scattering of the load-depth curves suggests a heterogeneous microstructure both before and after annealing. The maximum penetration depth varied between 5 and 20 µm for as-processed materials, which corresponds to a Vickers indentation diagonal of 35–140 µm. For the annealed sample, the indentation diagonals range from 7 to 35 µm. While these indentation sizes are much larger than the corresponding ligament sizes (which are 0.5 and 1.6 µm before and after annealing, respectively), the indentation results are nonetheless scattered. The pronounced spatial variation of the indentation behavior most likely arises from the hierarchical pore structures that form due to the relatively long pack cementation time (15 h) of route C. This means that micropores with an average size of approximately 6 μm coexist with the nanopores, which was caused by the multiphase structure of the pack cemented material (i.e., Al phase also forms beside Al_2_Cu) [18]. Such large pores can promote softer behavior for some indentations during indentation measurements, which manifests as a higher penetration depth. Moreover, there may be both lateral and in-depth variations in the bond strength between the ligaments, contributing to inhomogeneous indentation behaviors.

**Figure 8:**
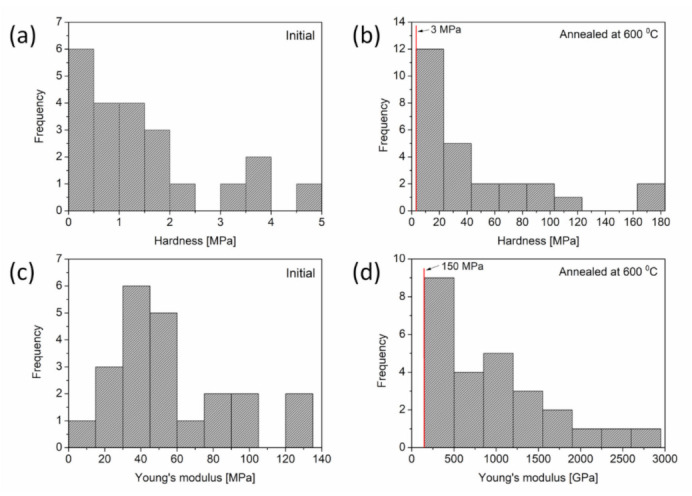
(**a**,**b**) Distributions of hardness and (**c**,**d**) Young’s modulus obtained by nanoindentation measurements conducted on Cu foams processed by (**a**,**c**) route C and (**b**,**d**) its counterpart annealed at 600 °C for 5.5 h after dealloying.

Figure 8 shows a plot of the distributions of hardnesses and Young’s moduli that were established from the load-depth indentation curves for Cu foams processed by route C, as well as its counterpart material that was annealed at 600 °C for 5.5 h after dealloying. The hardness and Young’s modulus have broad distributions, which is in accordance with the large variations in the load-depth curves. Even if there is a large scattering in the mechanical behavior of the materials, the heat treatment certainly yields a substantial increase in both hardness and Young’s modulus, as shown in Figure 6b. Namely, the mean hardness increased from 1.5 to 45 MPa, and the Young’s modulus increased from 56 to 990 MPa as a result of thermal annealing at 600 °C for 5.5 h. This observation suggests that the strengthening effects from ligament coalescence were greater than softening effects due to a reduction in the defect density inside the ligaments.

### 3.2. Influence of Oxidizing Heat Treatment on the Microstructure and Electrochemical Performance

**Figure 9:**
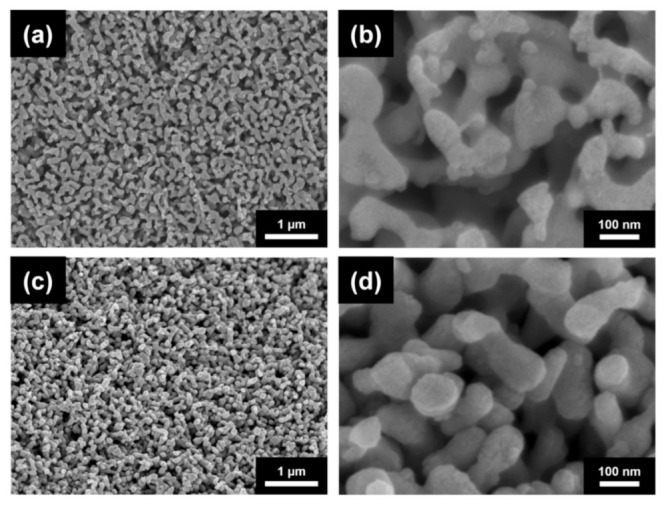
SEM images of Cu nanofoams oxidized at (**a**,**b**) 110 °C for 30 min and (**c**,**d**) at 140 °C for 30 min.

**Figure 10:**
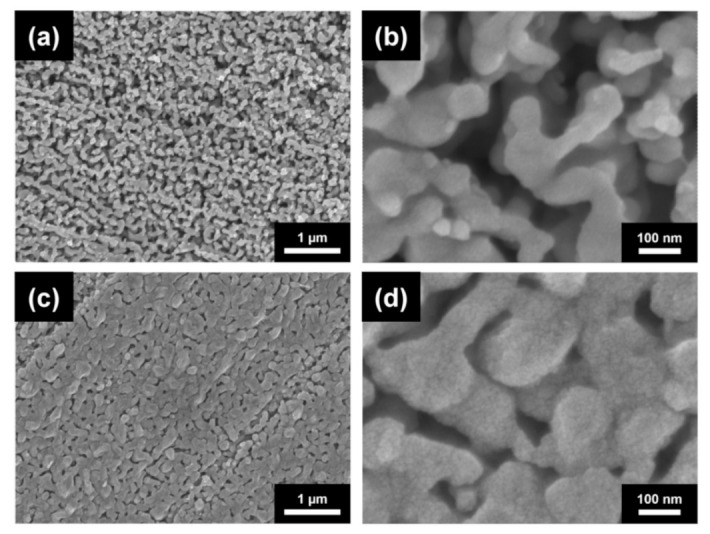
SEM images of Cu nanofoams oxidized at (**a**,**b**) 170 °C for 30 min and (**c**,**d**) 200 °C for 30 min.

Cu foams processed by dealloying were subsequently annealed under oxidizing atmospheres to investigate changes in the microstructure and electrochemical behaviors. The initial sample was processed by route A without annealing after dealloying. The average ligament size in the as-processed nanoporous Cu foam was approximately 160 nm which differs slightly from the value determined for the other sample used in the annealing processes performed in inert atmospheres. This difference in ligament sizes may be caused by the fact that the dealloying processes were performed in different research laboratories in Korea and Hungary, thus slight differences between the processing conditions cannot be excluded. In addition, the microstructures of the pack cemented disks used for dealloying in Korea and Hungary might be slightly different. Annealing in air was conducted for 30 min to grow oxidation layers for use as anode active material in Li-ion batteries. This additional heat treatment resulted in the increase of ligament size ranging from 177, 183, 210, to 260 nm sizes for 110, 140, 170, and 200 °C, respectively. The SEM images in Figure 9 and Figure 10 show representative examples of the microstructures of Cu foams annealed in air at different temperatures.

**Figure 11:**
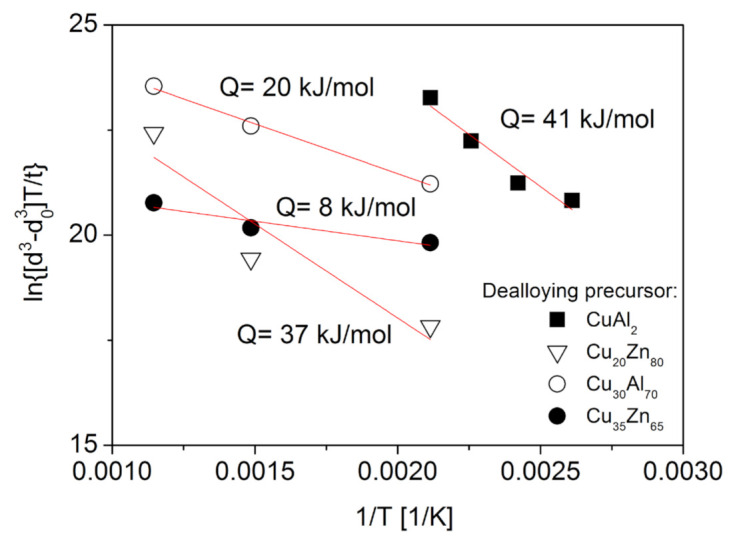
Plot of $ln\left\{\left[d^{3}-d_{0}^{3}\right]T/t\right\}$ versus 1/*T* to estimate the activation energy of the causal mechanism of ligament coarsening for dealloyed Cu foams annealed under an oxidizing atmosphere. Solid squares indicate materials processed by route A from CuAl_2_, open down triangles reflect materials dealloyed from Cu_20_Zn_80_ by using 5 wt.% HCl at RT for 72 h, solid circles indicate materials dealloyed from Cu_30_Al_70_ by using 85 wt.% H_3_PO_4_ at RT for 72 h, and open circles reflect materials dealloyed from Cu_35_Zn_65_ by using 5 wt.% HCl at RT for 72 h. The data for the latter three types of precursor alloy were taken from Reference [43].

Assuming that ligament coarsening under an oxidizing atmosphere can be described by Equations (1) and (2), $ln\left\{\left[d^{n}-d_{0}^{n}\right]T/t\right\}$ versus 1/*T* was plotted in Figure 11. In this evaluation, *n* = 3 was used since former studies (e.g., [43]) have shown that the ligament growth was caused mainly by the thickening of the oxide layer via Cu atom diffusion through the oxide layer. The data in Figure 11 are reasonably fitted by a straight line, the slope of which provides an estimate for the activation energy of the underlying mechanism for ligament growth. This provided an activation energy of about 41 kJ/mol that is approximately half of the value determined for materials annealed at high temperatures under an inert atmosphere (see Section 2.1). Similarly low activation energies were determined for the growth of oxide layers on the surface of Cu in a previous study of bulk materials [44], claiming an activation energy of 40–60 kJ/mol over the temperature range of 300 to 500 °C. The relatively low activation energy was explained by Cu migration along the grain boundaries of the oxide layer, specifically from the bottom of the layer to the surface, which served as the underlying process for the growth of the oxide layer. In this study, surface oxide layer growth most likely serves as the dominant factor for ligament coarsening in the Cu foams, thus resulting in a much lower activation energy than for materials annealed in an inert gas atmosphere.

A previous study also investigated ligament growth in three types of dealloyed Cu foams during oxidation for 0.5 h at temperatures of 200, 400, and 600 °C [43]. Three foams with porosities between 65% and 80% were processed by dealloying from Cu_20_Zn_80_ and Cu_35_Zn_65_ precursor alloys by using 5 wt.% HCl at room temperature for 72 h or from Cu_30_Al_70_ alloy using 85 wt.% H_3_PO_4_ at room temperature for 72 h. Subsequent annealing at 400 and 600 °C for 0.5 h transformed the whole ligaments from Cu to CuO. Figure 11 shows a plot of $ln\left\{\left[d^{3}-d_{0}^{3}\right]T/t\right\}$ versus 1/*T* plot for these three samples. The analysis yielded an activation energy between 8 and 37 kJ/mol. The large difference between these activation energies can be attributed to the distinct morphologies and ligament sizes of the dealloyed Cu foams, which may originate from different precursor compositions and dealloying solutions [43]. For these three foams, the ligament sizes varied between 60 and 100 nm. Moreover, the three Cu foam types may have different defect structures in the oxide layer. Defects such as planar fault, dislocations, and voids and cracks can facilitate diffusion. Thus, a higher defect density can result in very low (8–20 kJ/mol) activation energies for ligament coarsening during oxidizing heat treatments.

**Figure 12:**
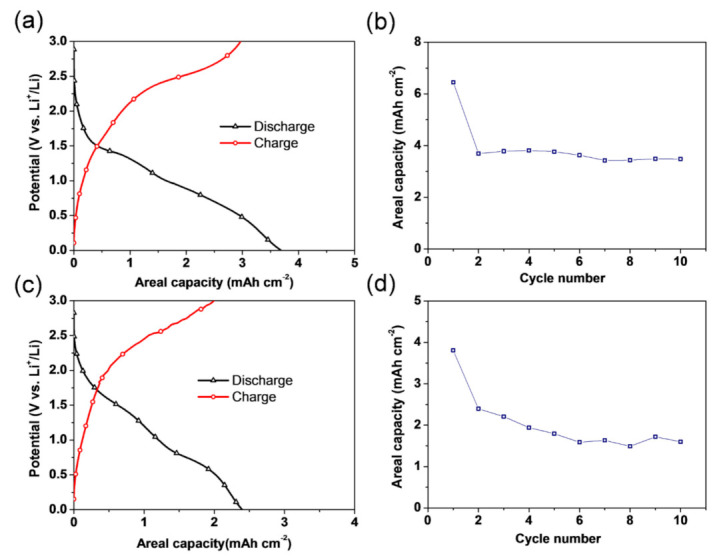
Voltage profiles of Cu foams oxidized at (**a**) 170 °C and (**c**) 200 °C, as well as a comparison of the areal capacity of Cu foam anodes that were oxidized at (**b**) 170 °C and (**d**) 200 °C at 1 mA/cm^2^ in 0.01–3.0 V.

Figure 12 shows the electrochemical properties of CuO/Cu_2_O/Cu foam anodes at a current density of 1 mA/cm^2^ over 0.01–3 V, where the Cu foam and CuO/Cu_2_O oxidation layers act as a current collector and active materials, respectively. The typical voltage profiles for both anodes prepared at different oxidation temperatures of 170 °C and 200 °C are displayed in Figure 12a,c. The discharge and charge profile shapes of both CuO/Cu_2_O/Cu foam anodes are similar to those reported for other CuO or Cu_2_O anode materials [45,46]. Figure 12b,d show the cycle performance of the CuO/Cu_2_O/Cu foam anodes oxidized at 170 °C and 200 °C.

Despite the general importance of evaluating the gravimetric or volumetric capacity, this study focused on the areal capacity of the CuO/Cu_2_O/Cu foam anode because of the difficulties in accurately measuring the surface area of nanoporous Cu foams and the corresponding mass of each active material in the CuO/Cu_2_O/Cu foam anode. The CuO/Cu_2_O/Cu foam anode that was oxidized at 170 °C has far superior cyclic performance and considerably higher stability than those of CuO/Cu_2_O/Cu foam anodes that were oxidized at 200 °C. Specifically, cells with CuO/Cu_2_O/Cu foam anode oxidized at 200 °C occasionally have voltage and capacity drops during cycling. This behavior arises from the decrease of the specific surface area due to ligament coarsening and the fact that higher temperatures are more favorable for CuO formation, which proceeds through the following reactions [47]:

(3) $$4\mathrm{Cu}+O_{2}\to 2{\mathrm{Cu}}_{2}O$$

(4) $$2{\mathrm{Cu}}_{2}O+O_{2}\to 4\mathrm{CuO}$$

Thus, the formation of CuO reduces the Cu_2_O layer, making these oxide active materials unstable during cycling. These electrochemical results demonstrate the need to further develop Cu nanofoams to enable the controlled, stable growth and formation of a Cu oxide layer on the Cu nanofoam surface, which would ultimately advance Cu nanofoams for practical uses in Li-ion battery applications.

## 4. Conclusions and Future Research Directions

This study provides new insights into the influence of heat treatment on the microstructure, defect density, mechanical behavior, and electrochemical performance of Cu nanofoams processed by dealloying, based on data available in the literature. Moreover, new results are presented and analyzed to reveal the processes during the thermal annealing of Cu foams. The following conclusions are drawn:

1. Heat treatment under inert atmospheres at temperatures between 300 and 800 °C results in significant ligament coarsening. Annealing at the lowest temperature of 300 °C increased the ligament size by only 20–30% versus non-annealed samples, even after 70 h of annealing time. Nonetheless, samples annealed at 800 °C had 700% greater ligament sizes even if the duration of heat treatment was only 5 h. The activation energy of ligament size growth was about 89–103 kJ/mol, suggesting that coarsening is controlled by fast diffusion either on the surface or along lattice defects.
2. The density of in-grown lattice defects, such as dislocations and twin faults, in the ligaments decreased significantly during annealing at 600 °C. Softening effects arising from the defect structures were overwhelmed by strengthening caused by the coalescence of the ligaments. Thus, heat treatments considerably improved the hardness and elastic modulus of materials.
3. Annealing at low temperatures (110–200 °C) for short (0.5 h) times under oxidizing atmospheres also moderately increased the ligament size. The activation energy of this coarsening process was only about 41 kJ/mol, which is close to the activation energy value of Cu diffusion along the grain boundaries in surface oxide layers. This mechanism is necessary for the growth of the oxide layer.
4. Electrochemical analyses demonstrated that oxidized Cu nanofoams can be considered a potential candidate as the anode material in high-performance Li-ion batteries. The CuO/Cu_2_O/Cu foam anode that was oxidized at 170 °C showed superior cycling stability than the CuO/Cu_2_O/Cu foam anode oxidized at 200 °C. Furthermore, the discharge and charge behaviors of both the CuO/Cu_2_O/Cu foam anodes are similar to those reported for other CuO or Cu_2_O anode materials.

To the best of our knowledge, a quantitative description of the effects of porosity on the yield strength, hardness, and elastic modulus of Cu nanofoams prepared by dealloying is not available. This might be due to the small sizes and weak mechanical strengths of samples that hinder conventional mechanical testing. By comparison, this study demonstrated that annealing is a suitable method to significantly improve the hardness of dealloyed foams. Thus, we recommend that future studies should generate foams with different porosities by using a combination of dealloying and heat treatments to establish correlations among the pore volume fraction and mechanical properties of Cu nanofoams. Moreover, we also recommend studying the microstructural coarsening in dealloyed Cu foams in which the active surface layer is not Cu oxide but another material, such as tin or silicon. Finally, it is also worth studying the effects of annealing on the microstructure and performance of dealloyed foams of composition other than Cu.

## Figures and Tables

**Table 1:** Ligament sizes of Cu foams processed and heat-treated under different conditions before and after thermal annealing.

Processing Conditions	Heat Treatment Conditions	Ligament Size before Annealing [nm]	Ligament Size after Annealing [nm]
Route A *	400 °C for 6 h	105 ± 6	125 ± 6
Route B **	300 °C for 70 h	640 ± 60	770 ± 50
Route B	800 °C for 5 h	640 ± 60	5180 ± 880
Route C ***	300 °C for 70 h	740 ± 50	980 ± 60
Route C	600 °C for 5.5 h	540 ± 20	1620 ± 80
Route C	800 °C for 5 h	740 ± 50	4220 ± 360

* Route A: Pack cementation at 800 °C for 6 h, homogenization at 700 °C for 9 h, and then at 500 °C for 6 h under an Ar atmosphere, dealloying in an aqueous solution of 2 wt.% HCl at 45 °C for 12 h [22]. ** Route B: Pack cementation at 800 °C for 15 h, homogenization at 700 °C for 9 h, and then at 500 °C for 6 h under an Ar atmosphere, dealloying in an aqueous solution of 5 wt.% HCl at 90 °C for 12 h. *** Route C: Pack cementation at 800 °C for 15 h, homogenization at 700 °C for 9 h, and then at 500 °C for 6 h under an Ar atmosphere, dealloying in an aqueous solution of 10 wt.% HCl at 90 °C for 12 h.

**Table 2:** The activation energy and the correlation coefficient obtained for fitting of $ln\left\{\left[d^{n}-d_{0}^{n}\right]T/t\right\}$ versus 1/*T* with the exponent values of *n* = 3 and 4. In the analysis, the Cu foams with the ligament sizes between 540 and 740 nm were used (see Table 1).

Exponent *n*	Activation Energy [kJ/mol]	Correlation Coefficient of Fitting
3	89 ± 5	0.99496
4	103 ± 11	0.99458

**Table 3:** Changes in the crystallite size and defect densities during annealing of Cu foams processed by dealloying under different conditions.

Processing Conditions	Crystallite Size [nm]	Dislocation Density [10^14^ m^2^ ]	Twin FaultProbability [%]
Route A *	18 ± 4	4 ± 1	2.1 ± 0.2
Route A + annealing at 400 °C for 5.5 h	142 ± 17	6 ± 1	1.0 ± 0.1
Route C **	60 ± 7	11 ± 2	1.1 ± 0.1
Route C + annealing at 600 °C for 5.5 h	90 ± 10	0.5 ± 0.2	0.1 ± 0.1

* Route A: Pack cementation at 800 °C for 6 h, homogenization at 700 °C for 9 h, and then at 500 °C for 6 h, etching in an aqueous solution of 2 wt.% HCl at 45 °C for 12 h [22]. ** Route C: Pack cementation at 800 °C for 15 h, homogenization at 700 °C for 9 h, and then at 500 °C for 6 h in an Ar atmosphere, etching in an aqueous solution of 10 wt.% HCl at 90 °C for 12 h.

## Data Availability

The evaluated data presented in this study are available in the tables of this paper. The raw measured data of this study are available on request from the corresponding author.
